# Reactive Blends of Thermoplastic Starch and Epoxidized Natural Rubber with Chlorhexidine Gluconate Compatibilizer and Antimicrobial Agent

**DOI:** 10.3390/polym18202494

**Published:** 2026-10-12

**Authors:** Pimlapat Nanjai, Pornchai Rachtanapun, Sarinthip Thanakkasaranee, Nuttapol Tanadchangsaeng, Patnarin Worajittiphon, Kittisak Jantanasakulwong

**Affiliations:** 1 Department of Packaging Technology, Faculty of Agro-Industry, Chiang Mai University, Chiang Mai 50100, Thailand; pimlapatinging@gmail.com (P.N.); pornchai.r@cmu.ac.th (P.R.); sarinthip.t@cmu.ac.th (S.T.); 2 Center of Excellence in Agro Bio-Circular-Green Industry (Agro BCG), Chiang Mai University, Chiang Mai 50100, Thailand; 3 College of Biomedical Engineering, Rangsit University, Pathumthani 12000, Thailand; nuttapol.t@rsu.ac.th; 4 Department of Chemistry, Faculty of Science, Chiang Mai University, Chiang Mai 50200, Thailand; patnarin.w@cmu.ac.th

**Keywords:** starch, chlorhexidine gluconate, biodegradation of plastics, sustainable agriculture

## Abstract

In this study, reactive blending of thermoplastic starch (TPS), chlorhexidine gluconate (CHG), and epoxidized natural rubber (ENR) was performed. CHG was incorporated to improve compatibility via crosslinking and antimicrobial properties, whereas ENR was added to improve the flexibility and interfacial compatibility of the TPS/CHG/ENR blends. TPS was prepared from gelatinized cassava starch and glycerol (70/30 wt/wt%) at 70 °C. CHG was incorporated into TPS at concentrations of 0.5, 1, 2, 5, and 10% (w/w). The TPS/CHG (0.5–10%) samples were melt-blended with ENR (90/10 wt/wt%) at 135 °C. FTIR confirmed the hydrogen bonding of TPS/CHG, whereas the opening of the epoxy ring formed a C–O–C reaction with the –NH_2_ groups of CHG. In the TPS/CHG5/ENR blend, the tensile strength increased to 13.8 MPa from the crosslinking reaction. The water contact angle increased from 30° (TPS) to 76° (TPS/CHG10/ENR) after 10 min due to the hydrophobicity of the materials and the crosslinking reaction. The TPS/CHG2/ENR sample exhibited inhibition zones against both *Staphylococcus aureus* and *Escherichia coli*, whereas the TPS/CHG10/ENR sample exhibited antifungal activity against *Rhizopus oligosporus*.

## 1. Introduction

Plastics have been an integral part of modern human life for decades. Their commercial deployment commenced during the Second World War and rapidly proliferated globally in less than a century. Most conventional plastics are synthesized from petroleum-based materials, which render them relatively inexpensive, highly processable, and amenable to property modification through diverse manufacturing and compounding techniques. Although these materials offer substantial functional and economic advantages, their widespread use has precipitated significant environmental and public health concerns [1]. Increasing scientific and industrial attention has shifted toward the development and utilization of biodegradable plastics as sustainable alternatives.

Biopolymers have emerged as promising candidates for replacing petroleum-derived plastics owing to their intrinsic biodegradability and broad compatibility with other biomaterials. Various biopolymeric substances, including starch, have been investigated for their potential as functional replacements of conventional plastics, particularly for packaging applications. Among these, thermoplastic starch (TPS) has emerged as one of the most widely adopted and extensively studied bioplastic systems [2].

TPS is produced by incorporating a plasticizer into starch under combined heat and shear conditions, which enables the mobility of polymer chains and facilitates melt processing. Among the available plasticizers, glycerol is the most widely used and cost-effective option for TPS fabrication, owing to its ability to interact strongly with starch molecules through extensive hydrogen bonding. However, TPS has several inherent drawbacks, including inadequate mechanical strength, poor water resistance, and limited antimicrobial performance. To overcome these constraints, TPSs are often modified via reactive blending with various bio-based materials to enhance their overall properties [3].

Epoxidized natural rubber (ENR) is a chemically modified natural rubber in which epoxide groups are introduced into polymer chains. ENR can be prepared through the coagulation of natural rubber using peroxy acids such as performic acid. The degree of epoxidation is controlled by factors such as the peroxy acid concentration, reaction temperature, and reaction duration. The introduction of epoxide groups led to an increase in the glass transition temperature (*T*_g_) of ENR compared to unmodified natural rubber, with an increase of approximately 1 °C for every 1 mol% of epoxidation. This change in *T*_g_ significantly influences the dynamic mechanical behavior of the material [4]. ENR exhibits excellent elasticity and toughness, high mechanical strength, good oil resistance, strong adhesive properties, and high damping performance. The presence of epoxide groups increases the polarity of the rubber, thereby enhancing its compatibility with polar polymers in polymer blend systems. These properties originate from the highly polar epoxide groups, which are randomly distributed along the natural rubber backbone. Consequently, ENR is regarded as a promising material for use as a toughening agent in a variety of polymer matrices [4].

Chlorhexidine gluconate (CHG) is an antimicrobial agent that exhibits activity against Gram-negative and Gram-positive bacteria, mycobacteria, fungi, and endotoxins. The molecular structure of CHG comprises two 4–chlorophenyl rings and two biguanide groups connected by a central hexamethylene chain, which allows for interactions with other molecules through chemical bonding. In TPS systems, CHG acts as a crosslinking agent, compatibilizer, and antimicrobial agent. The incorporation of CHG into TPS improves tensile properties, hardness, and water resistance of the blends, which is attributed to crosslinking interactions between the NH_2_ groups of CHG and the hydroxyl (–OH) groups of starch [5]. Previous studies have demonstrated the beneficial effects of CHG in TPS, particularly in improving mechanical and antimicrobial properties [3,5]. However, these studies have mainly focused on TPS/CHG systems, whereas the incorporation of ENR introduces an additional elastomeric phase and creates a distinct challenge in achieving sufficient compatibility while maintaining the antimicrobial function of CHG. Thus, the present study extends the TPS/CHG blend to a ternary TPS/CHG/ENR blend and systematically investigates the influences of CHG concentration on the interactions between the TPS and ENR phases, phase morphology, mechanical properties, water resistance, thermal stability, rheological behavior, and antimicrobial activity. The novelty of this work therefore lies in the development and systematic characterization of a CHG-containing TPS/ENR ternary blend, rather than simply demonstrating the effect of CHG on TPS.

Therefore, this study investigated the improvement in TPS properties through reactive blending with ENR using CHG as both a crosslinking and an antimicrobial agent in TPS/ENR blends. The morphologies, reactivities, mechanical properties, water resistances, thermal stabilities, and antimicrobial activities of the TPS/CHG/ENR blends were characterized. CHG played a multifunctional role in TPS/ENR blends as a compatibilizer, crosslinker, and antimicrobial, providing new biodegradable active materials for agriculture, medicine, and packaging applications.

## 2. Materials and Methods

### 2.1. Materials

Cassava starch with an amylose/amylopectin content of 17:83 w/w (Dragon Fish brand with a moisture content of 11% total weight and a molecular weight of 1.34 × 108 g/mol) was purchased from Tong Chan, registered in an ordinary partnership, Bangkok, Thailand. Glycerol was purchased from Union Science Co., Ltd. (Chiang Mai, Thailand). CHG was purchased from Tong Chemical Co., Ltd. (Nonthaburi, Thailand). ENR with 25 mol% epoxidation (ENR25) was purchased from Muangmai Katree Co., Ltd. (Phuket, Thailand).

### 2.2. Sample Preparation

The TPS was prepared using cassava starch and glycerol in a 70/30 ratio (w/w) and 500 mL of distilled water in a water bath at 70 °C for 30 min using a stirrer at 500 rpm. To prepare TPS/CHG, cassava starch premixed with glycerol was blended with 0.5, 1, 2, 5, and 10% (w/w) CHG and then dried in a hot air oven at 50 °C for 24 h [3]. The mixing ratios are listed in Table 1.

The prepared TPS/CHG samples with different CHG contents (90 wt%) were mixed with ENR (10 wt%) using a two-roll mill at 135 °C for 20 min. The samples obtained were then compressed into sheets using a hot compression machine at 135 °C for 10 min.

### 2.3. Fourier-Transform Infrared (FTIR) Spectroscopy

Fourier-transform infrared (FTIR) spectroscopy (FTIR-4700, Jasco Corp., Tokyo, Japan) was used to examine chemical interactions. The samples were fabricated as thin sheets and molded at 135 °C for 10 min prior to analysis. FTIR spectra were recorded in the wavenumber range 600–4000 cm^−1^ with a spectral resolution of 4 cm^−1^.

### 2.4. Tensile Properties

Tensile tests were conducted following JISK6251–7 using a universal testing machine (H1KS; Hounfield Testing Equipment, Surrey, UK). The initial grip separation was set to 10 mm, and the crosshead speed was maintained at 10 mm/min. Test specimens with widths, lengths, and thicknesses of 5 mm, 30 mm, and 1 mm, respectively, were used. Each measurement was performed using five independent specimens (*n* = 5). All specimens were conditioned at 25 °C and 50 ± 2% relative humidity for 24 h. The maximum tensile force and elongation at break were obtained from the tests.

### 2.5. Scanning Electron Microscopy (SEM)

The morphologies of the fractured surfaces were observed using scanning electron microscopy (SEM, JSM-IT200, JEOL Co., Ltd., Tokyo, Japan). The specimens were cryo-fractured by immersion in liquid nitrogen, and then the fractured surfaces of the samples were extracted by immersing the samples in toluene at room temperature (25 °C) for 24 h. The extracted sample surfaces were coated with a thin layer of gold via sputtering. The SEM observations were performed under vacuum at an accelerating voltage of 15 kV.

### 2.6. Shore A Hardness Test

The Shore A hardness was evaluated using a Shore durometer (E2-D, Imada Co., Ltd., Toyohashi, Japan). The samples were prepared as sheets by hot extrusion molding at 135 °C for 3 min. Hardness was measured at five different locations on each specimen, and measurements were averaged for each specimen.

### 2.7. Contact Angle

The water contact angle was determined using a drop-shaped analyzer (DSA30E, Krüss Co., Ltd., Hamburg, Germany). Samples with dimensions of 20 × 20 × 1 mm (width × length × thickness) were used. Images of the water droplet on the sample surface were captured at 1 min intervals over a period of 10 min, and the contact angle was calculated through image analysis.

### 2.8. Swelling Ratio

The water swelling behavior was evaluated using specimens with dimensions of 1.5 × 1.5 × 0.1 cm (width × length × thickness). The samples were dried at 50 °C for 24 h and subsequently immersed in 50 mL of distilled water at 25 °C for 1 h. The swelling ratio was calculated using Equation (1) [6]

(1) $$\mathrm{Swelling ratio}\;(\%)=\frac{\mathrm{Ws}-\mathrm{Wd}}{\mathrm{Wd}}\times 100$$

where *W*_s_ and *W*_d_ are the swollen and dry weights, respectively.

### 2.9. Solubility Measurement

The solubility percentage was determined using specimens with dimensions of 1.5 × 1.5 × 0.1 cm (width × length × thickness). The samples were dried at 60 °C for 24 h. Each specimen was then immersed in 50 mL of distilled water and shaken continuously at 25 °C for 24 h. After immersion, the solution was filtered to collect undissolved residues. The filter paper containing the residue was dried in a convection oven at 50 °C for 24 h. The solubility was calculated using Equation (2) [7]

(2) $$\mathrm{Solubility}\;(\%)=\frac{W0-W2}{W0}\times 100$$

*W*_0_ and *W*_2_ are the initial and dry weights of the filtered sample, respectively.

### 2.10. Thermogravimetric Analysis (TGA)

Thermogravimetric analysis was conducted under a nitrogen atmosphere using a thermogravimetric analyzer (TGA/DSC3+, Mettler-Toledo STARe, Greifensee, Switzerland). Approximately 9–12 mg of each sample was subjected to heating from 30 °C to 500 °C at a constant heating rate of 25 °C/min.

### 2.11. Rheological Properties

The complex dynamic viscosity (η*) was determined using a rheometer. Test specimens were fabricated as circular disks by compression molding at 135 °C for 10 min, with a thickness of 1 mm and a diameter of 25 mm. Rheological measurements were carried out over an angular frequency range of 0.1–1000 rad/s at a fixed strain of 10% and a constant temperature of 150 °C.

### 2.12. Antimicrobial Activity

Antimicrobial performance was assessed using the agar disk diffusion technique. The samples were evaluated against two bacterial strains, *Escherichia coli* and *Staphylococcus aureus*, and a fungal strain, *Rhizopus oligosporus*. The TPS/ENR film without CHG was used as the reference polymer film. For comparison with freely available CHG, pure CHG was directly dropped onto the surface of the CHG-free TPS/ENR film and used as a CHG positive reference antifungal agent. In contrast, CHG incorporation into the TPS/ENR matrix at concentrations of 0.5–10 wt% was evaluated as an integrated antimicrobial additive. Pure amoxicillin was used as a positive reference antibacterial agent. These reference samples were included to distinguish the antimicrobial activity of CHG incorporated within the polymer matrix from that of externally applied CHG and to provide a reference for antibacterial activity. The microorganisms were incubated at 37 °C for 10 h, and the microbial concentration was adjusted to 10^8^ CFU/mL. The specimens, prepared by extrusion at 135 °C for 10 min with a diameter of 5 mm and a thickness of 1 mm, were placed on agar plates and incubated at 37 °C for 48 h. Antimicrobial activity of the samples was quantified by measuring the diameter of the inhibition zones. Three independent specimens were tested for each formulation (*n* = 3).

### 2.13. Statistical Analysis

One-way ANOVA was performed using SPSS software version 17 (SPSS, Armonk, NY, USA) to analyze the results. Differences (*p* < 0.05) were estimated using Duncan’s test.

## 3. Results and Discussion

### 3.1. Reaction Mechanism

**Figure 1:**
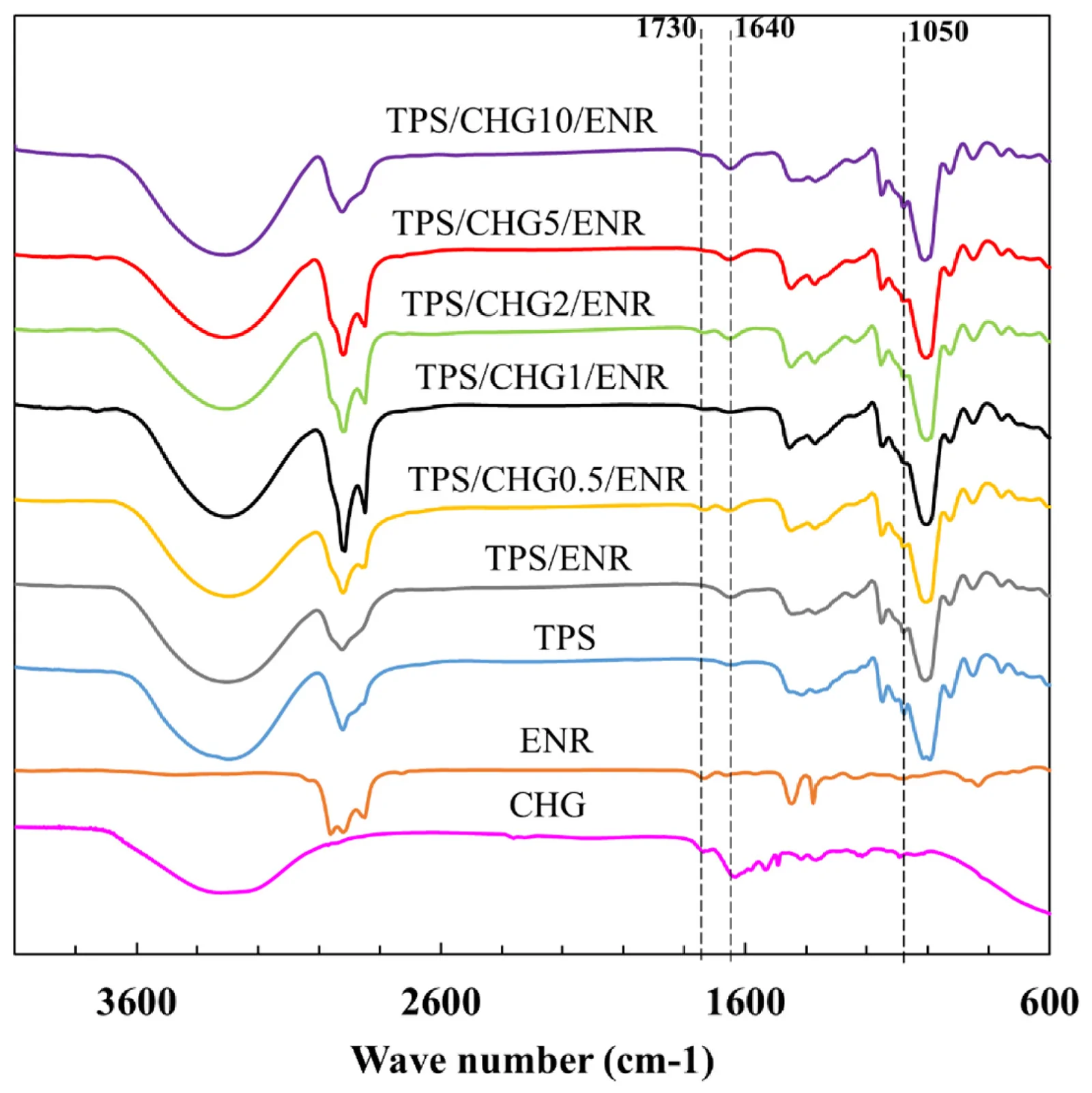
Fourier-transform infrared spectra (FTIR) of chlorhexidine gluconate (CHG), epoxidized natural rubber (ENR), thermoplastic starch (TPS), TPS/ENR, and TPS/ENR/CHG composites with 0.5–10 wt% CHG contents.

**Figure 2:**
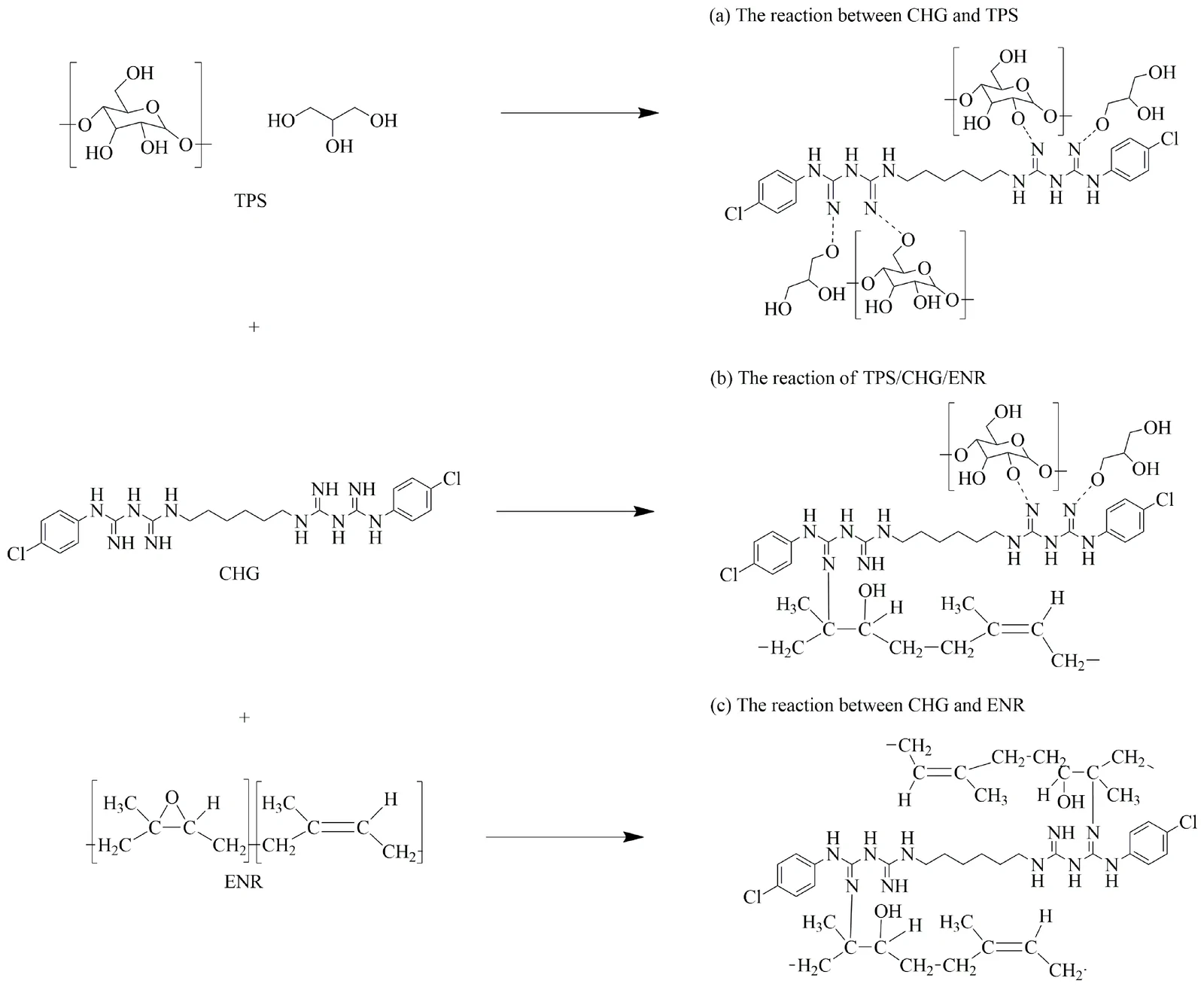
Expected reaction mechanisms of TPS/ENR/CHG blend (**a**) the expected reaction between CHG and TPS, (**b**) the expected reaction between CHG and ENR, and (**c**) the expected reaction between CHG and ENR.

Figure 1 presents the FTIR spectra of TPS, ENR, CHG, and the TPS/CHG/ENR composites with different CHG contents. Neat TPS exhibited a broad absorption band in the range of 3200–3500 cm^−1^, corresponding to O–H stretching vibrations from starch and glycerol hydroxyl groups, which contain extensive hydrogen bonding in the TPS matrix. The characteristic bands observed at 1020–1080 cm^−1^ were attributed to C–O–C and C–O stretching vibrations of the starch glycosidic structure, confirming that the polysaccharide backbone remained intact after thermoplastic processing [8,9,10,11]. The FTIR spectrum of ENR showed a characteristic absorption band at 870–910 cm^−1^. which was assigned to the epoxide ring vibration, a distinctive feature of ENR. For neat CHG, characteristic absorption bands were observed at 1640–1650 cm^−1^, corresponding to N–H bending and C=N stretching vibrations, respectively [12]. The TPS/ENR blend exhibited the characteristic FTIR features of both components. The slight broadening of the O–H stretching region at 3200–3500 cm^−1^ indicated intermolecular interactions between TPS and ENR, suggesting enhanced compatibility of the blended system [13]. The incorporation of CHG into the TPS/ENR distinctly changed the FTIR spectra. The O–H stretching band at 3200–3500 cm^−1^ in TPS/CHG/ENR composites became broader and of higher intensity compared to TPS and TPS/ENR. This indicated enhanced hydrogen bonding interactions among the starch, glycerol, and CHG molecules. The absorption band at 1640 cm^−1^ of the CHG N–H bending vibrations combined with the peak of the TPS/ENR matrix, while a new band appeared at 1730 cm^−1^ due to new C=O stretching vibrations, indicating a small amount of CHG (0.5%). CHG contains –NH groups and –Cl end groups in its structure, whereas the –NH groups showed strong interactions with the –OH groups of TPS [3,5,12]. Then, when the ENR was added, the remaining CHG NH groups reacted with the epoxy groups of ENR. Reaction between the –NH groups and epoxy groups has also been reported [14]. The intensity of the 1730 cm^−1^ absorption decreased with increasing CHG content and disappeared at 5 wt% CHG. This spectral behavior was consistent with the proposed reaction between the –NH groups of CHG and the epoxy groups of ENR and suggested a reduction in the interaction between the CHG –NH groups and the TPS –OH groups [14] (Figure 2). This behavior confirmed the reaction between the –NH groups of CHG and the epoxy groups of ENR. The peak at 1730 cm^−1^ was again present with increasing intensity at 1645 cm^−1^, and a small peak at 1560 cm^−1^ in TPS/CHG10/ENR was due to the peak of excessive CHG content (10 wt%). The observed spectral changes suggest that the combined molecular interactions among TPS, CHG, and ENR contribute to the formation of an interconnected TPS/CHG/ENR structure, which was consistent with the changes in the properties.

### 3.2. Mechanical Properties

**Figure 3:**
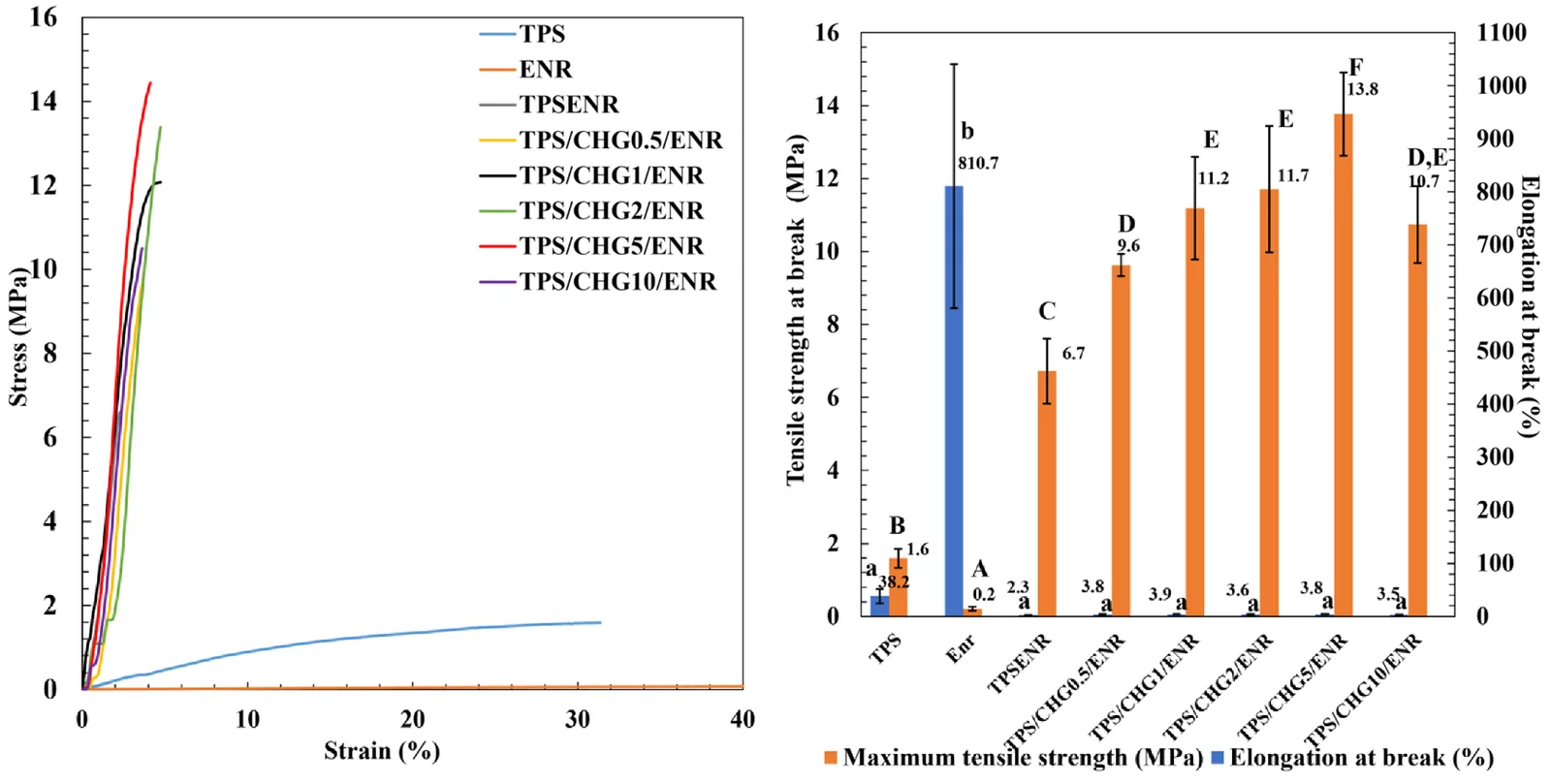
Stress–strain curves of TPS, TPS/ENR, and TPS/ENR/CHG blends with 0.5–10 wt% CHG contents, *n* = 5. The mean values of the elongation at break (a–b lowercase letters) and the maximum tensile strength (A–F uppercase) significantly differ (*p* < 0.05).

Figure 3 shows the tensile properties of TPS/ENR composites containing different amounts of CHG. TPS exhibits low tensile strength and low elongation at break owing to its brittle structure and the low intermolecular bonding between the polymer chains. ENR exhibited high elongation at break but low tensile strength, reflecting the characteristics of rubbery materials with these properties. In the TPS/ENR blend, the elongation at break significantly decreased, while the tensile strength increased from 1.6 to 6.7 MPa compared with neat TPS owing to the interactions between the TPS and ENR phases. The incorporation of CHG at 0.5–5 wt% (based on TPS) into the TPS/ENR system resulted in a continuous increase in tensile strength with increasing CHG content. The TPS/ENR/CHG5 composite exhibited the highest tensile strength (13.8 MPa) because CHG enhanced interfacial adhesion and acted as a crosslinking agent between TPS and ENR through interactions and reactions among the CHG amino groups (–NH), the starch hydroxyl groups (–OH), and the ENR epoxy groups [5,12,15]. However, the TPS/CHG10/ENR blend showed decreased tensile strength because of the additional CHG, which induced crosslinking inside ENR to form agglomerates [5,12,14]. Agglomeration has been widely reported in composite materials with excessive amounts of crosslinking additives, which lead to poor interfacial adhesion [16,17,18]. The elongation at break values of the TPS/ENR/CHG blends were lower than those of neat ENR. The crosslinking reaction inside the TPS and TPS/ENR interfaces enhanced the tensile strength but decreased the flexibility of the blend, whereas crosslinking inside the ENR phase reduced the tensile strength because of the ENR agglomeration effect. At 2 wt% CHG, CHG-induced interactions and reactions between the TPS matrix and the ENR phase resulted in improved stress transfer and increased tensile strength. At 5 wt% CHG, the expected crosslinking within the ENR phase increased the tensile strength, while excessive CHG (10 wt%) reduced the tensile strength due to excessive crosslinking within the ENR phase.

### 3.3. Morphology

**Figure 4:**
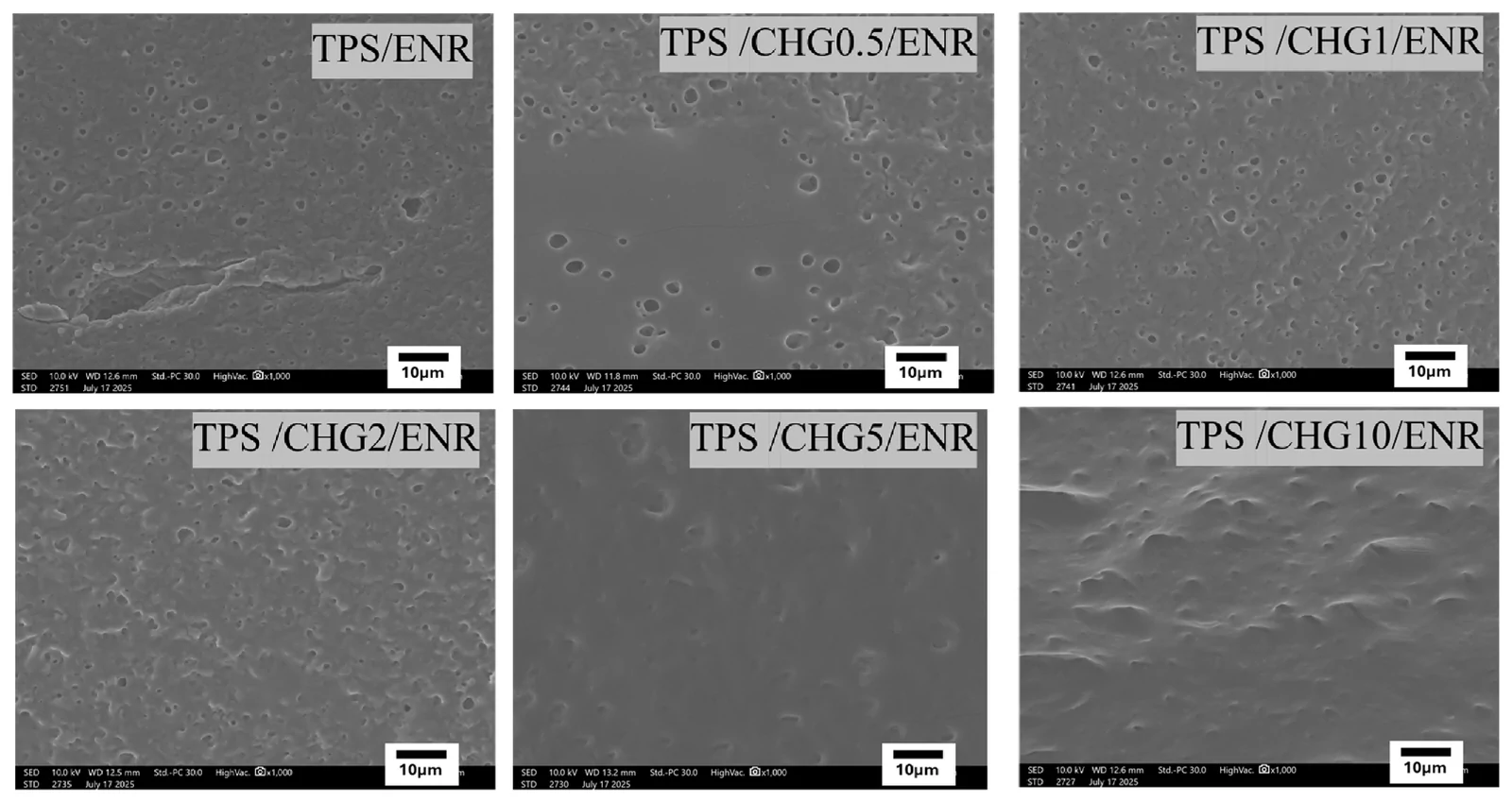
Scanning electron microscopy (SEM) images of the extracted fracture surface by toluene of TPS/ENR and TPS/ENR/CHG blends with different CHG contents.

Figure 4 illustrates the fracture surface morphology of the TPS/ENR composites containing different amounts of CHG after extraction with toluene. In the TPS/ENR sample, numerous large, irregular voids were observed throughout the fractured surface owing to the removal of ENR by the solvent. The TPS/ENR composite with 0.5–2 wt% CHG also exhibited a phase-separated morphology with ENR-extracted voids. The CHG contents of the 1–2 wt% samples showed a uniform pore distribution due to the high interfacial reaction between the –NH groups of CHG and epoxy groups of ENR. TPS/CHG5/ENR showed a large number of pores, while ENR aggregation was indicated by the excessive amount of CHG formed by crosslinking inside the ENR phase. TPS/CHG10/ENR presented large ENR particles without holding the extracted ENR because ENR formed high crosslinking inside the ENR phase via the CHG reaction [15]. The network formed inside the ENR phase was not dissolved by toluene and presented a surface without holes or large ENR particles.

### 3.4. Shore A Hardness

**Figure 5:**
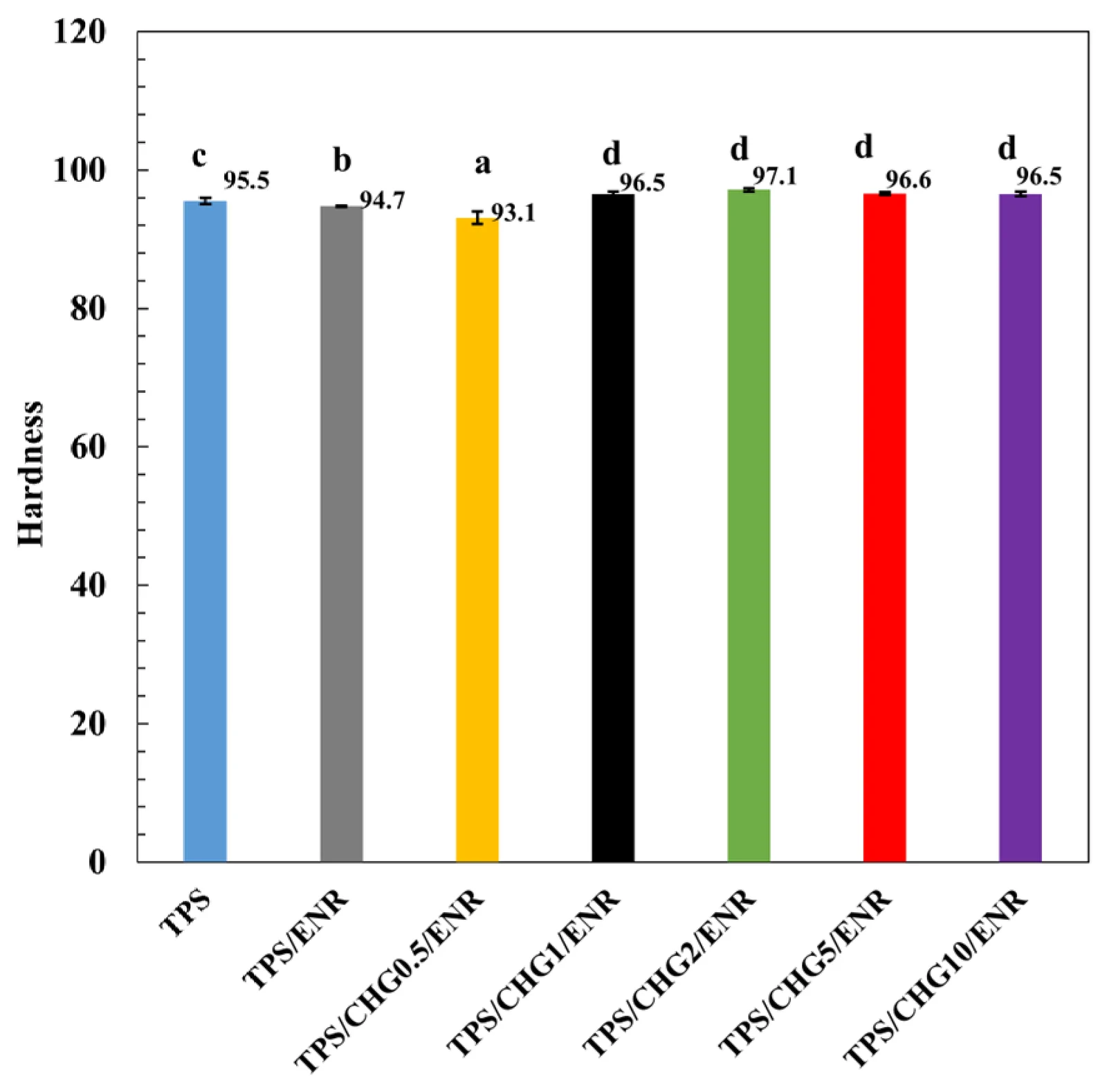
Shore A hardness of TPS, TPS/ENR, and TPS/ENR/CHG composites with 0.5–10 wt% CHG contents, *n* = 5. The mean values indicated by the different (a–d) lowercase superscript letters significantly differ (*p* < 0.05).

Figure 5 shows the hardness test results for TPS, TPS/ENR, and TPS/CHG/ENR with CHG contents of 0.5, 1, 2, 5, and 10 wt%. The hardness of TPS was 95.5, whereas that of ENR was 94.7. The TPS/CHG0.5/ENR sample showed a decrease in hardness to 93.1 compared to that of TPS/ENR because of the better compatibility between TPS and ENR via the CHG compatibilizer. Increasing the CHG content to 1–10 wt% enhanced the hardness of the TPS/ENR to approximately 96–97 owing to the interfacial crosslinking and crosslinking network inside the ENR phase. The increase in hardness can be explained by the enhanced polymer network density resulting from crosslinking among the hydroxyl groups (–OH) of TPS, amino groups (–NH) of CHG, and epoxy groups of ENR, which restricted the mobility of the polymer chains within the matrix [18,19]. Crosslinking inside the ENR phase also increased the stiffness of the material, which was related to the hardness of TPS/CHG/ENR. The CHG-improved interactions and reactions between TPS and ENR enhanced the composite structural homogeneity, leading to increased deformation resistance and a significant increase in hardness. Reactions between TPS and CHG, as well as between ENR and CHG, have been previously reported [3,5,12].

### 3.5. Contact Angle

**Figure 6:**
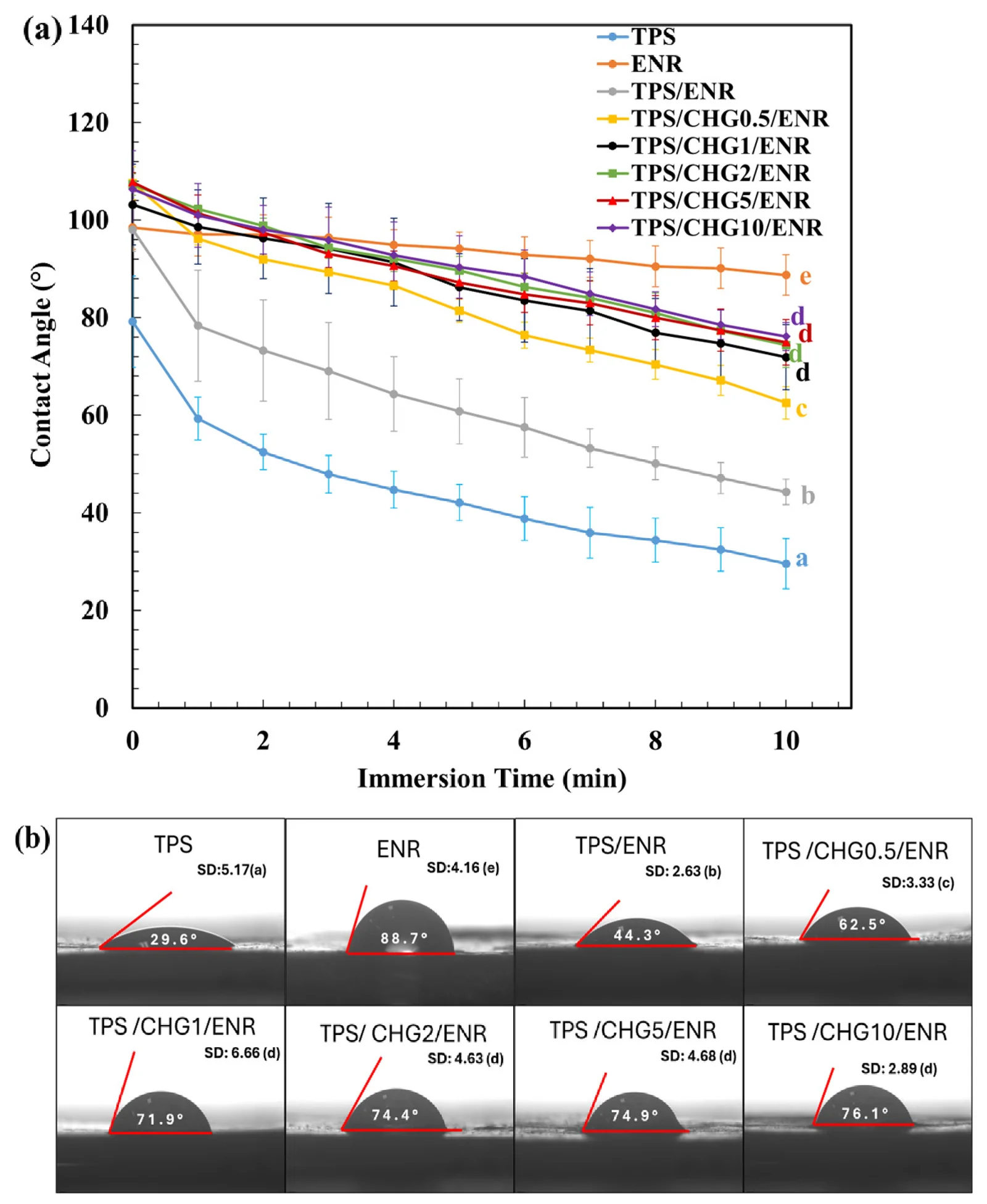
Water contact angles of TPS, ENR, TPS/ENR, and TPS/ENR/CHG blends with 0.5–10 wt% CHG contents: (**a**) plots of samples and (**b**) values at 10 min. Different lowercase letters indicate significant differences among the mean contact angle values at 10 min (*p* < 0.05).

Figure 6 shows the water contact angle behavior of the TPS/ENR composites containing different amounts of CHG. TPS exhibited a low water droplet contact angle, with a decreasing trend of 29° after 10 min of immersion because of the hydrophilic surface of TPS. ENR showed a markedly high contact angle of 98°, which slowly decreased to 88.7° after 10 min of immersion, indicating strong hydrophobicity of the ENR phase. The TPS/ENR blend exhibited a contact angle of 98°, which decreased to 44.3° due to the hydrophilic surface of the TPS matrix. The addition of CHG to TPS/ENR increased the contact angle with increasing CHG content (0.5, 1, 2, 5, and 10 wt%) to 62.5, 71.9, 74.4, 74.9, and 76.1°, respectively. All samples exhibited a gradual decrease in water contact angles over time from 0 to 10 min. TPS showed the most rapid decline because of its hydrophilicity, whereas ENR and the TPS/CHG10/ENR blend maintained high contact angles owing to their high hydrophobicity and crosslinking, respectively. The contact angles of the TPS/CHG/ENR blends increased with increasing CHG content because of the increase in TPS/ENR reaction trough CHG. These results were related to the mechanical property improvements and reduced extractability of ENR in the tensile and morphological analyses, respectively. An improvement in the water contact angle due to the crosslinking of TPS has also been reported [3,13]. CHG induced interactions and reactions among the TPS, CHG, and ENR components, resulting in improved dispersion of the rubber phase within the TPS matrix (Figure 4). The improved dispersion of the hydrophobic ENR phase increased the hydrophobic character of the exposed surface, thereby contributing to the increase in water contact angle. This behavior was distinguished from swelling and solubility, which presented the bulk water uptake and dissolution resistance of the material, respectively.

### 3.6. Solubility and Swelling

**Figure 7:**
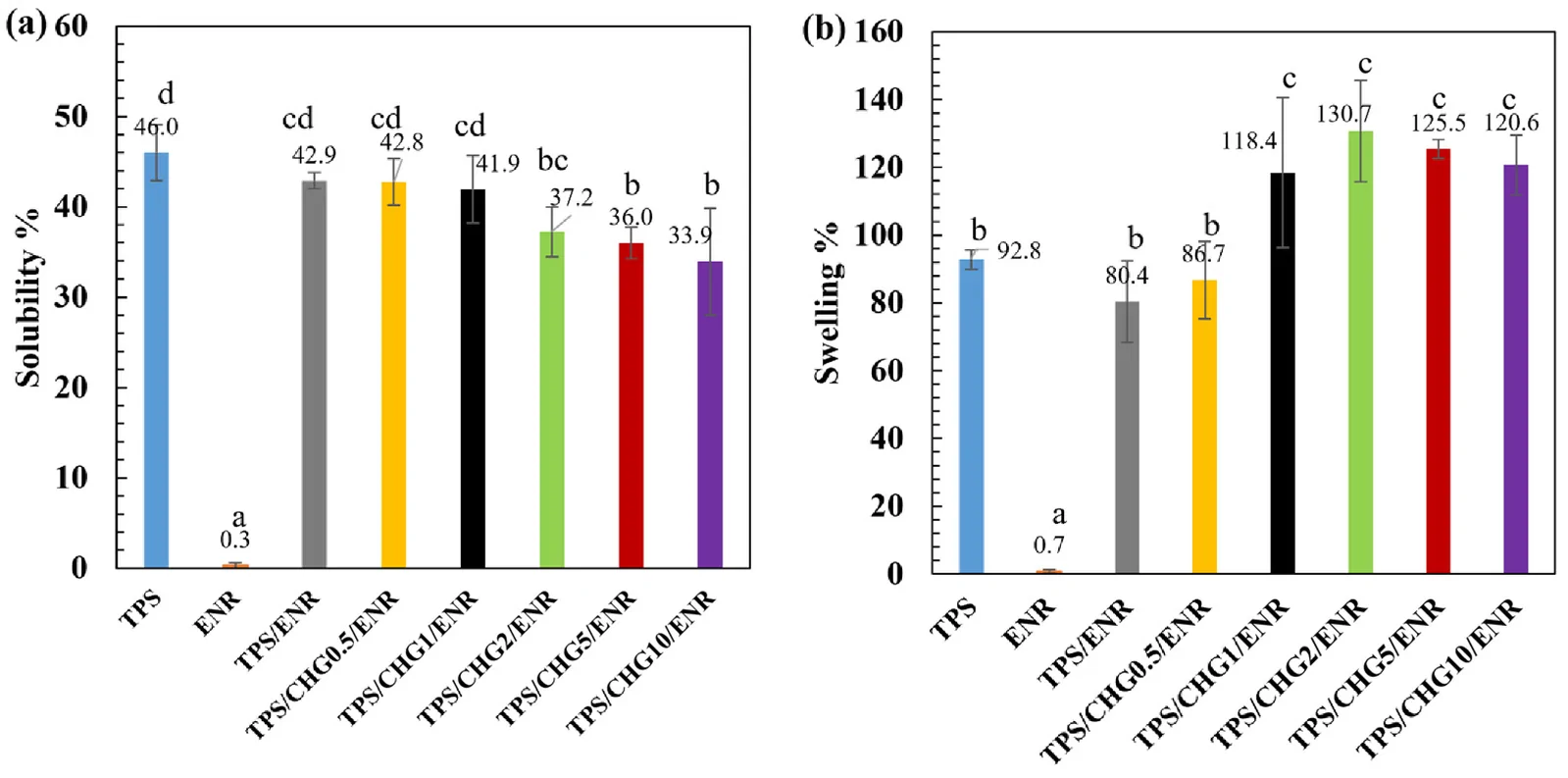
Percentage of composites with 0.5–10 wt% CHG contents (*n* = 3): (**a**) solubility of TPS, ENR, TPS/ENR, and TPS/ENR/CHG composites; (**b**) swelling of TPS, ENR, TPS/ENR, and TPS/ENR/CHG composites. The mean values indicated by the different lowercase superscript letters are significantly different (*p* < 0.05).

The solubility and swelling behaviors of the TPS/ENR blends with different CHG contents were investigated to evaluate the effect of CHG-induced interactions on the water-related properties and network structure of the blends. The solubility and swelling results of TPS, ENR, TPS/ENR, and TPS/CHG/ENR with 0.5–10 wt% CHG are shown in Figure 6. Neat TPS exhibited a high solubility (46.0%) because of the strongly hydrophilic nature of the starch–glycerol matrix, whereas ENR showed negligible solubility (0.3%) because of its hydrophobic character. The incorporation of ENR into TPS reduced the solubility to 42.9% of that of TPS owing to the partial suppression of starch dissolution by the rubber phase. Increasing CHG content gradually decreased the solubility of the TPS/CHG/ENR blends. The solubilities of the TPS/CHG/ENR blends with 0.5, 1, 2, 5, and 10 wt.% CHG decreased significantly to 42.8, 41.9, 37.2, 36.0, and 33.9%, respectively (Figure 7a). CHG effectively restrained the extractability of the TPS chains through the formation of a highly crosslinked network to enhance the network stability and dissolution resistance of the network structure [5,19].

The swelling behavior exhibited a different trend than solubility (Figure 7b). Neat TPS exhibited a swelling ratio of 92.8%, whereas ENR exhibited low swelling at 0.7%. The TPS/ENR blend exhibited a swelling value of 80.4% owing to the contribution of the hydrophobic rubber phase in the TPS matrix. The swelling ratios of the TPS/CHG/ENR blends gradually increased with the addition of 0.5, 1, and 2 wt% CHG to 86.7, 118.4, and 130.7%, respectively. This increase indicates enhanced water uptake, a more open polymer network structure, and a chain movement network of TPS/ENR through the CHG crosslinking reaction. At high CHG contents of 5 and 10 wt%, the swelling slightly decreased to 125.5 and 120.6%, respectively, because the high crosslink density restricted chain expansion and swelling. Large-aggregate ENR obtained by CHG 5 and 10% crosslinking inside the ENR phase also remained undissolved in water and restricted the chain mobility of the sample to reduce solubility and swelling, respectively [12,20]. The different trends between swelling and contact angle were attributed to the different mechanisms measured by these tests. At 0.5–2 wt% CHG, the CHG-induced interactions facilitated structural rearrangement and allow for greater water uptake, resulting in increased swelling, whereas at 5–10 wt% CHG, the highly cross-linked ENR phase restricts chain expansion and water uptake, leading to reduced swelling. In contrast, the continuous decrease in solubility with increasing CHG content increased structure connectivity, which reduced the dissolution of polymer chains in water.

### 3.7. Thermal Stability

**Figure 8:**
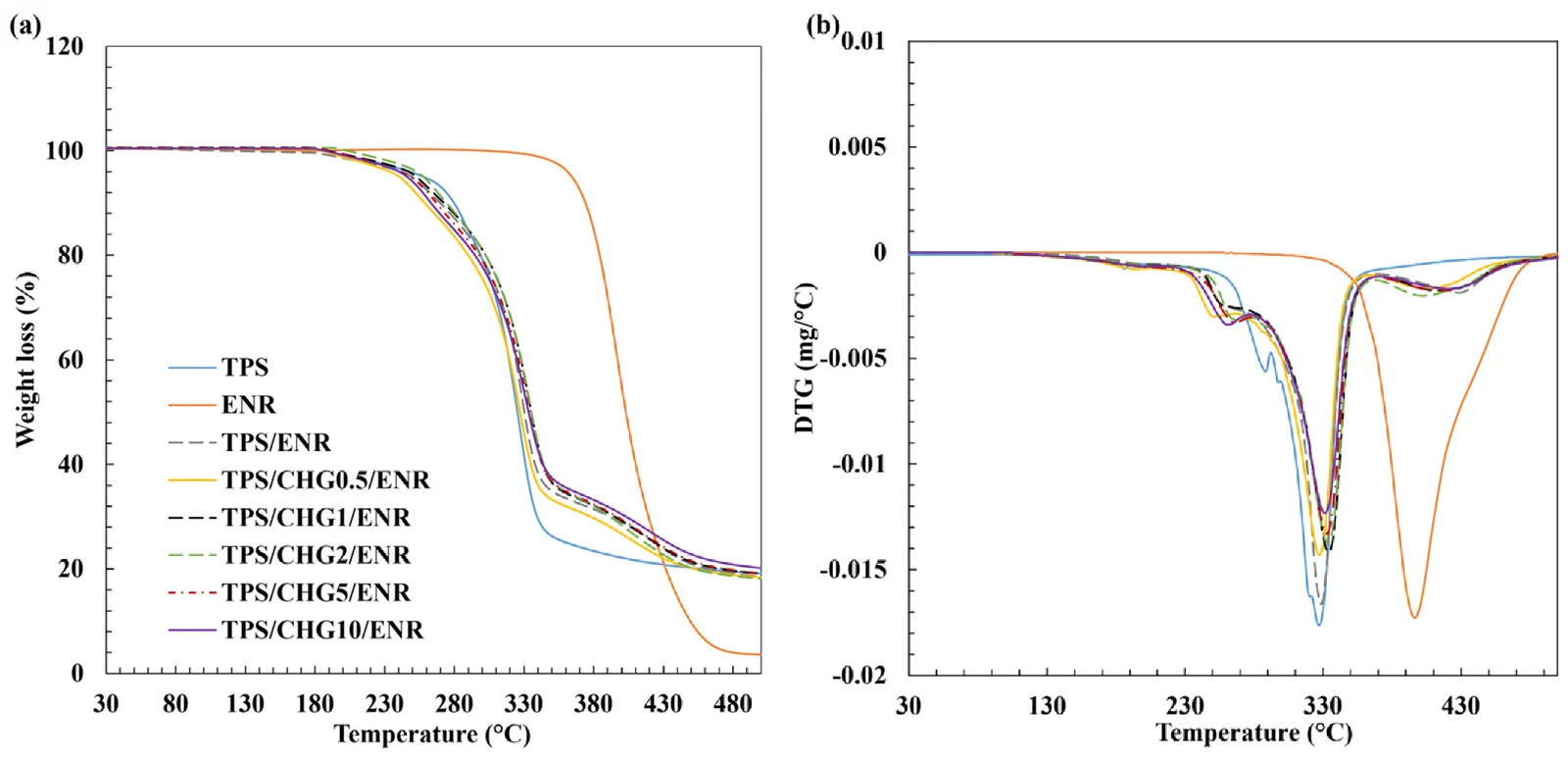
Thermal analysis of TPS, ENR, TPS/ENR, and TPS/ENR/CHG composites with 0.5–10 wt% CHG contents: (**a**) TGA curves; (**b**) DTG curves.

Figure 8 shows the TGA and derivative thermographic analysis (DTG) curves of TPS, ENR, TPS/ENR, and TPS/ENR/CHG blends with 0.5, 1, 2, 5, and 10 wt% CHG. TPS exhibited a major thermal degradation step at 320–330 °C (Table 2) due to the decomposition of the starch matrix and plasticizer, while neat ENR showed a single dominant degradation peak in a higher temperature region around 400–430 °C, indicating its high thermal stability [21,22,23,24,25]. The TPS/ENR blend displayed the two overlapping degradation peaks of the TPS and ENR thermal decomposition phases [26]. CHG incorporation modified the thermal degradation behavior of the TPS/ENR blend. The main degradation peak of the TPS slightly shifted to higher temperatures, while the residual mass at high temperatures (above 450 °C) increased gradually with CHG content. These results indicated an improvement in the thermal stability of the TPS/CHG/ENR blends compared to those of neat TPS and the unmodified TPS/ENR blend. The enhanced thermal resistance was attributed to the formation of additional chemical crosslinking through hydrogen bonding interactions between the TPS matrix and the amino groups of CHG and the chemical reaction between the amino groups of CHG and the epoxy groups of ENR. This crosslinking restricted chain mobility and delayed the thermal decomposition process [19]. The increase in char residue with CHG content indicated the formation of a highly compact and thermally stable TPS–CHG–ENR network structure [26,27,28,29].

### 3.8. Rheological Properties

**Figure 9:**
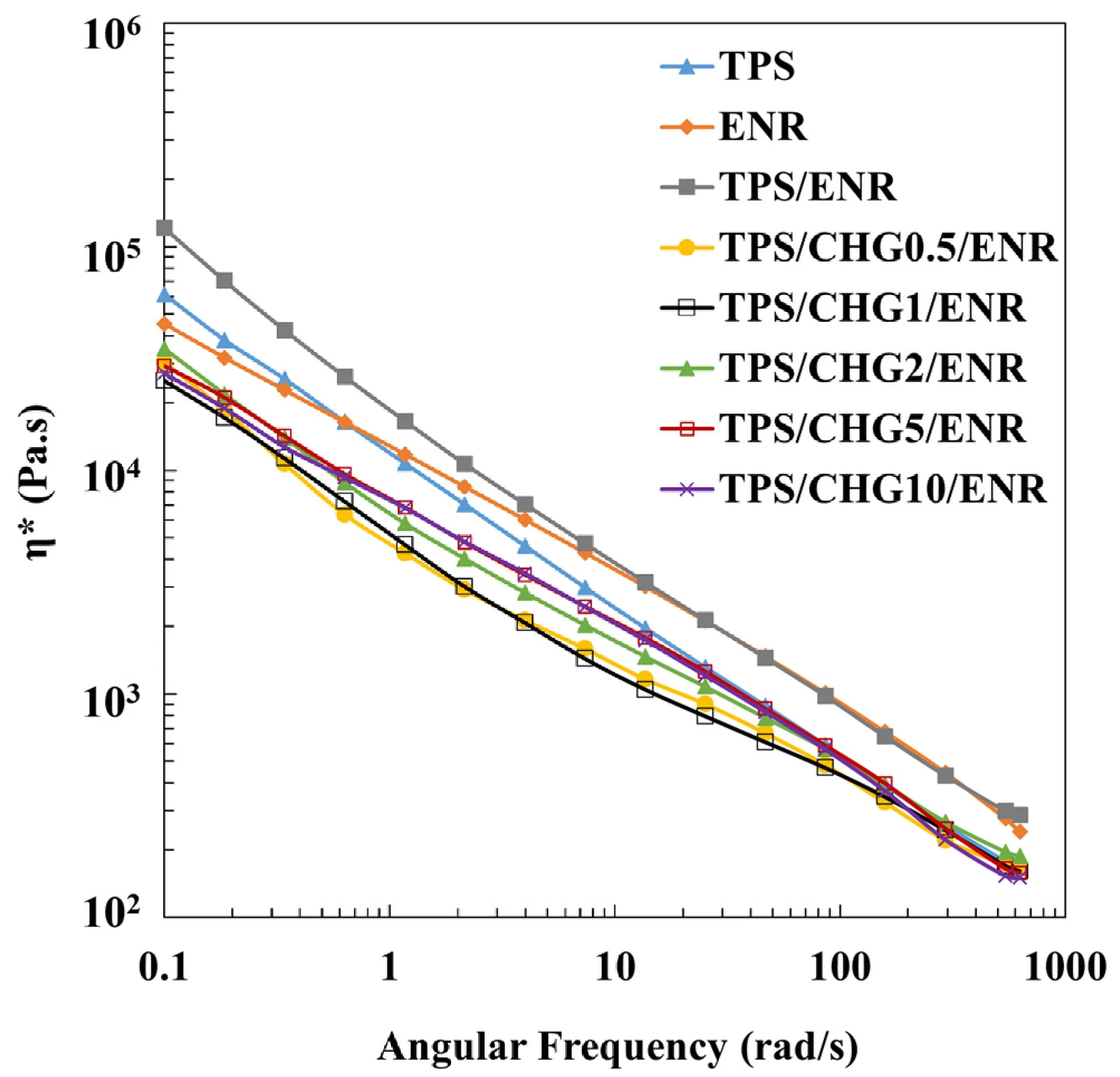
Complex melt viscosity of TPS, ENR, TPS/ENR, and TPS/ENR/CHG composites with 0.5–10 wt% CHG contents at 150 °C.

Figure 9 shows the complex viscosity (η*) of TPS, ENR, TPS/ENR, and TPS/ENR/CHG blends with 0.5, 1, 2, 5, and 10 wt% CHG. All the samples exhibited a decrease in viscosity with frequency, which is common for polymer melts and polymer blends, owing to the alignment and disentanglement of polymer chains under shear [30,31]. TPS showed relatively high viscosity at low frequencies owing to its high molecular association and limited chain mobility owing to extensive hydrogen bonding among the starch chains [8,32]. The TPS/ENR blend displayed a higher viscosity than neat TPS across most of the frequency range, indicating the influence of the entangled ENR phase, which increased the resistance to flow owing to its elastomeric nature. ENR exhibited a lower viscosity than TPS/ENR at low frequencies because of its elastomer characteristics and lower molecular entanglement compared to the TPS matrix [33]. The complex viscosities of the TPS/CHG0.5/ENR and TPS/CHG1/ENR blends generally decreased, suggesting that the plasticizing and compatibilizing effect of CHG promoted chain mobility and reduced internal friction between the TPS and ENR phases. The enhanced compatibility resulted from intermolecular interactions and possible weak crosslinking between CHG and the TPS and ENR functional groups, which reduced the resistance to flow under low shear conditions. The initial decrease in complex viscosity with CHG addition was related to CHG-induced structural changes in the ENR phase, which resulted in smaller and more dispersed rubber domains and facilitated melt flow. The complex viscosity of the TPS/CHG/ENR blends increased with increasing CHG content, which was attributed to increased network interactions and reactions at TPS/ENR interface through CHG. High CHG content also promoted crosslinking inside ENR phase and increased the viscosity. These networks restricted the chain mobility of the TPS/CHG/ENR blends [19,34]. CHG effectively decreased the melt viscosity of the TPS/ENR system, which improved the processability of melt processing operations and flow characteristics through compatibilization [12,34,35]. This trend differed from the tensile strength behavior because the tensile strength was influenced not only by melt viscosity but also by rubber-phase dispersion and interfacial interactions between TPS and ENR, which improved stress transfer within the matrix.

### 3.9. Antimicrobial Activity

**Figure 10:**
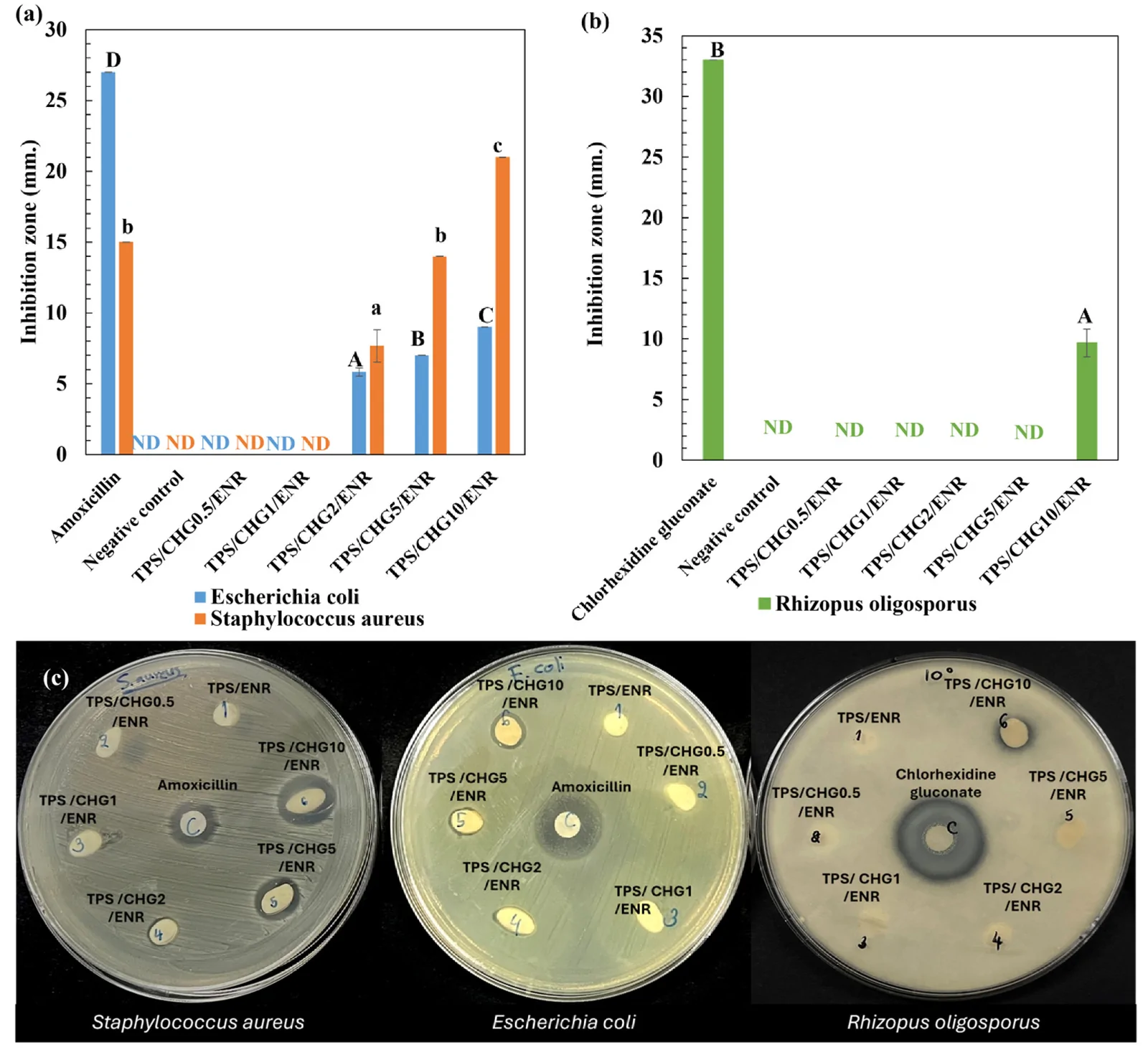
Antimicrobial activity of TPS/ENR and TPS/ENR/CHG composites with 0.5–10 wt% CHG contents (*n* = 3): (**a**) inhibition zones against bacteria; (**b**) inhibition zones against fungi; (**c**) visual images of inhibition zones showing microbial activity against *Staphylococcus aureus*, *Escherichia coli*, and *Rhizopus oligosporus*. ND indicates not detected. The mean values indicated by A–D uppercase letters (*E. coli*), a–c lowercase letters (*S. aureus*), and A–B uppercase letters (*R. oligosporus*) are significantly different (*p* < 0.05).

The antimicrobial activity of the TPS/ENR blends with 0–10 wt% CHG was evaluated using the agar disk diffusion method against *E. coli*, *S. aureus*, and *R. oligosporus*. The inhibition zones and quantitative results are shown in Figure 10. For both bacteria, no inhibition zones were detected for the TPS/ENR and TPS/ENR blends with low CHG contents (0.5 and 1 wt%) because of insufficient CHG release at low loading levels. A small inhibition zone was observed for TPS/CHG2/ENR, indicating the onset of antibacterial effectiveness. An increase in the diameter of the inhibition zone was observed at higher CHG loadings. The TPS/CHG/ENR blends with 5 and 10 wt% CHG exhibited clear inhibition zones against both *E. coli* and *S. aureus*, confirming the concentration-dependent antimicrobial activity of CHG in the TPS/ENR matrix. In all formulations, *S. aureus* showed larger inhibition zones than *E. coli* because the Gram-positive *S. aureus* was more susceptible to CHG, consistent with the greater permeability of Gram-positive cell walls to cationic antiseptics [36]. In contrast, antifungal activity against *R*. *oligosporus* was observed only at the highest CHG loading (10 wt%). No inhibition zones were detected for the TPS/CHG/ENR blends with 0–5 wt% CHG, whereas inhibition zones were formed around the TPS/CHG10/ENR specimen. High CHG concentrations are required to suppress fungal growth compared to bacterial inhibition. The antimicrobial performance at elevated CHG content was attributed to the increased availability and diffusion of chlorhexidine molecules from the matrix into the surrounding agar medium. CHG exerted its antimicrobial activity by disrupting microbial cell membranes through electrostatic interactions, leading to leakage of intracellular components and cell death. The higher sensitivity of *S. aureus* relative to *E. coli* is related to previous reports describing the reduced susceptibility of Gram-negative bacteria owing to the protective outer membrane barrier [37].

## 4. Conclusions

The reactive blending of TPS/CHG/ENR led to the development of multifunctional crosslinking and antimicrobial activity. ENR enhanced the mechanical strength and surface hydrophobicity of TPS, whereas CHG promoted interfacial compatibilization through hydrogen bonding and epoxy–amine reactions by enabling a more compact and stable network structure. The optimum CHG content of 5 wt% provided the highest tensile strength, improved thermal stability, and reduced solubility, whereas higher CHG loadings (10 wt%) induced excessive crosslinking and brittleness. The TPS/CHG/ENR blends exhibited concentration-dependent antimicrobial activity with effective antibacterial performance at ≥2 wt% CHG and antifungal activity at 10 wt%. CHG reduced the melt viscosity and improved the processability of the TPS/ENR matrix, acting as both a crosslinking agent to improve the compatibility of the blends and an antimicrobial agent to provide antimicrobial properties to the blends. This reactive blending is an efficient strategy for enhancing the mechanical performance, water-related properties, and antimicrobial properties of TPS/ENR blends with strong potential for future development in antimicrobial packaging applications.

## Figures and Tables

**Table 1:** Composition of TPS blends with CHG.

Samples	TPS (wt/wt%)	CHG (wt/wt%)
TPS	100	0
TPS/CHG0.5	99.5	0.5
TPS/CHG1	99	1
TPS/CHG2	98	2
TPS/CHG5	95	5
TPS/CHG10	90	10

**Table 2:** Thermal decomposition characteristics of TPS, ENR, TPS/ENR, and TPS/ENR/CHG composites with 0.5–10 wt% CHG content.

Samples	1st Region			2nd Region			3rd Region			Residue at 500 °C (%)
	Range (°C)	Peak (°C)	Weight Loss (%)	Range (°C)	Peak (°C)	Weight Loss (%)	Range (°C)	Peak (°C)	Weight Loss (%)	
TPS	188.28–304.80	291.17	20.396	305.33–365.95	332.88	60.040	-	-	-	19.16
ENR	341.73–476.43	402.49	94.597	-	-	-	-	-	-	3.62
TPS/ENR	179.03–284.11	255.83	10.75	283.37–358.71	329.49	51.66	362.13–482.94	430.63	14.46	18.57
TPS/CHG0.5/ENR	237.69–276.38	252.96	11.58	277.28–362.48	333.19	52.00	363.14–491.90	417.82	13.26	18.40
TPS/CHG1/ENR	235.97–275.66	252.48	8.98	277.26–363.24	331.89	53.24	367.44–491.01	420.41	14.37	19.18
TPS/CHG2/ENR	233.23–283.21	258.57	12.77	283.48–363.51	332.50	53.14	363.77–471.70	416.90	12.90	18.21
TPS/CHG5/ENR	234.82–282.00	260.82	9.78	283.61–365.49	331.57	51.76	366.02–479.16	421.18	14.50	19.20
TPS/CHG10/ENR	228.33–282.17	256.53	12.76	282.70–366.92	331.03	48.78	368.49–481.38	368.49–481.38	14.53	20.22

## Data Availability

The original contributions presented in this study are included in the article. Further inquiries can be directed to the corresponding author.

## Abbreviations

The following abbreviations are used in this manuscript:

TPS	Thermoplastic starch
CHG	Chlorhexidine gluconate
ENR	Epoxidized Natural Rubber
